# The Mechanism of Ethylene Signaling Induced by Endophytic Fungus *Gilmaniella* sp. AL12 Mediating Sesquiterpenoids Biosynthesis in *Atractylodes lancea*

**DOI:** 10.3389/fpls.2016.00361

**Published:** 2016-03-23

**Authors:** Jie Yuan, Kai Sun, Meng-Yao Deng-Wang, Chuan-Chao Dai

**Affiliations:** Jiangsu Key Laboratory for Microbes and Functional Genomics, Jiangsu Engineering and Technology Research Center for Industrialization of Microbial Resources, College of Life Sciences, Nanjing Normal UniversityJiangsu, China

**Keywords:** *Atractylodes lancea*, endophytic fungi, sesquiterpenoids, ethylene, medicinal plants, tissue culture

## Abstract

Ethylene, the first known gaseous phytohormone, is involved in plant growth, development as well as responses to environmental signals. However, limited information is available on the role of ethylene in endophytic fungi induced secondary metabolites biosynthesis. *Atractylodes lancea* is a traditional Chinese herb, and its quality depends on the main active compounds sesquiterpenoids. This work showed that the endophytic fungus *Gilmaniella* sp. AL12 induced ethylene production in *Atractylodes lancea*. Pre-treatment of plantlets with ethylene inhibiter aminooxyacetic acid (AOA) suppressed endophytic fungi induced accumulation of ethylene and sesquiterpenoids. Plantlets were further treated with AOA, salicylic acid (SA) biosynthesis inhibitor paclobutrazol (PAC), jasmonic acid inhibitor ibuprofen (IBU), hydrogen peroxide (H_2_O_2_) scavenger catalase (CAT), nitric oxide (NO)-specific scavenger 2-(4-Carboxyphenyl)-4,4,5,5-tetramethylimidazoline-1-oxyl-3-oxide potassium salt (cPTIO). With endophytic fungi inoculation, IBU or PAC did not inhibit ethylene production, and JA and SA generation were suppressed by AOA, showing that ethylene may act as an upstream signal of JA and SA pathway. With endophytic fungi inoculation, CAT or cPTIO suppressed ethylene production, and H_2_O_2_ or NO generation was not affected by 1-aminocyclopropane-1-carboxylic acid (ACC), showing that ethylene may act as a downstream signal of H_2_O_2_ and NO pathway. Then, plantlets were treated with ethylene donor ACC, JA, SA, H_2_O_2_, NO donor sodium nitroprusside (SNP). Exogenous ACC could trigger JA and SA generation, whereas exogenous JA or SA did not affect ethylene production, and the induced sesquiterpenoids accumulation triggered by ACC was partly suppressed by IBU and PAC, showing that ethylene acted as an upstream signal of JA and SA pathway. Exogenous ACC did not affect H_2_O_2_ or NO generation, whereas exogenous H_2_O_2_ and SNP induced ethylene production, and the induced sesquiterpenoids accumulation triggered by SNP or H_2_O_2_ was partly suppressed by ACC, showing that ethylene acted as a downstream signal of NO and H_2_O_2_ pathway. Taken together, this study demonstrated that ethylene is an upstream signal of JA and SA, and a downstream signal of NO and H_2_O_2_ signaling pathways, and acts as an important signal mediating sesquiterpenoids biosynthesis of *Atractylodes lancea* induced by the endophytic fungus.

## Introduction

*Atractylodes lancea*, belonging to the Compositae family, is a traditional Chinese medicinal plant and is used as a main ingredient in many famous Chinese medicines. Sesquiterpenoids are the main active compounds in *A. lancea* and have medicinal efficacy against influenza, digestive disorders, rheumatic diseases, and night blindness (Wang et al., 2008). The quality of this herb depends on where it is cultivated. The plantlet grown in the Maoshan area of the Jiangsu Province is the geo-authetic medicinal plant (Ouyang et al., 2012), and is characterized by higher content of sesquiterpenoids (Ji et al., 2001). In recent years, the natural sources of *A. lancea* have been in sharp decline as they grow slowly and have been over exploited (Zhou et al., 2015). Although artificially cultivated sources of *A. lancea* ensure the production of this herb, the content of sesquiterpenoids is relatively low (Zhou et al., 2015). Currently, guaranteeing sesquiterpenoids content in *A. lancea* has become a hot topic. Endophytes play active roles in promoting plant growth and secondary metabolites accumulation (Wang et al., 2011; Ludwig-Müller, 2015). Our previous studies have shown that several endophytes, such as *Gilmaniella* sp. AL12, *Acinetobacter* sp. ALEB16, and *Pseudomonas fluorescens* ALEB7B, can establish symbiotic relationships with *A. lancea*, and also greatly promote sesquiterpenoids accumulation in the herb (Wang et al., 2012, 2015a; Zhou et al., 2015). How endophytes promote the accumulation of sesquiterpenoids in *A. lancea* is an intriguing issue.

Some works have been done to explain the phenomenon of the improving sesquiterpenoids accumulation in *A. lancea* caused by the endophytes (Wang et al., 2011, 2012, 2015a; Ren and Dai, 2012, 2013; Ren et al., 2013). Our previous studies demonstrated that AL12 can activate signals, such as nitric oxide (NO), hydrogen peroxide (H_2_O_2_), salicylic acid (SA) (Wang et al., 2011), jasmonic acid (JA) (Ren and Dai, 2012), brassinosteroid (Br) (Ren and Dai, 2013), and Calcium (Ca^2+^) (Ren et al., 2013), increasing the biosynthesis of sesquiterpenoids in *A. lancea*. Whether there are other signals involved in the signaling pathway for AL12-induced sesquiterpenoids accumulation of *A. lancea* is worthy of attention.

Ethylene (ET) is the first known gaseous phytohormone, and affects plant growth, development, and responses to environmental signals (Arc et al., 2013; Steffens, 2014; Bakshi et al., 2015; Wei et al., 2015). ET acted as an important signal and was involved in the production of β-thujaplicin (Zhao et al., 2004), lycopene (Liu et al., 2012), ginsenoside (Rahimi et al., 2015), and terpenoid (Arimura et al., 2007). We have mainly focused on the signals of JA, SA, NO, H_2_O_2_ in AL12-induced sesquiterpenoids accumulation of *A. lancea*, and did not pay attention to the ET signaling. The aims of this study are to investigate whether the endophytic fungus AL12 can induce ET generation, and to discuss the possible role of ET in sesquiterpenoids accumulation, and also to expound the possible relationship between ET and other signals. Furthermore, we compared the signaling pathways mediating sesquiterpenoids accumulation induced by endophytic fungi and endophytic bacterium. This work comprehensively demonstrated the signaling pathways of sesquiterpenoids biosynthesis and provided a theoretical basis for the industrialization of active compounds in *A. lancea*. And this work will provide a theoretical reference for the biosynthesis of other active compounds such as artemisinin, paclitaxel, menthol, glycyrrhizic acid, and ginseng saponin, and will help to further clarify plant-endophyte interactions.

## Materials and methods

### Plant material and growth conditions

Meristem cultures of *A. lancea* were established using tissue culture as previously described (Wang et al., 2012). Briefly, sterilized plantlets were grown in 50 mL Murashige and Skoog medium containing 30 g L^−1^ sucrose, 10% agar (w/v), 0.3 mg L^−1^ naphthaleneacetic acid, and 2.0 mg L^−1^ 6-benzyladenine in 150-mL Erlenmeyer flasks. When newborn axillary buds produced by the meristem cultures were sufficient, they were separated and transplanted into 50 mL Murashige and Skoog medium containing 30 g L^−1^ sucrose, 10% agar (w/v), and 0.25 mg L^−1^ naphthaleneacetic acid in 150-mL Erlenmeyer flasks. All media pH was adjusted to 6.0 before autoclaving at 121°C for 20 min. Plants were maintained in a growth chamber at 25/18°C day/night cycle, with a light intensity of 3400 lm/m^2^ and a photoperiod of 12 h, and were sub-cultured every 30 days.

### Endophytic fungi and inoculation

The fungal endophyte AL12 (*Gilmaniella* sp.) was isolated from *A. lancea*, cultured on potato dextrose agar, and incubated at 28°C for 5 days prior to starting any experiments (Wang et al., 2012). Thirty-day-old rooting plantlets were inoculated with 5-mm AL12 mycelial disks, which were placed near the plant caudexes on the medium. Meantime, controls were established using equal sized potato dextrose agar disks. All treatments were performed under sterile conditions and performed in triplicate.

### Chemicals and treatments

Aminooxyacetic acid (AOA), ibuprofen (IBU), paclobutrazol (PAC), catalase (CAT), 2-(4-Carboxyphenyl)-4,4,5,5-tetramethylimidazoline-1-oxyl-3-oxide potassium salt (cPTIO) were used as specific inhibitors or scavengers of ethylene, JA, SA, H_2_O_2_, NO synthesis, and they were obtained from Sigma-Aldrich (St. Louis, MO, USA). 1-aminocyclopropane-1-carboxylic acid (ACC), Sodium nitroprusside (SNP) were used as donors of ethylene and NO, respectively. JA, SA, hydrogen peroxide solution were obtained from Sigma-Aldrich (St. Louis, MO, USA). All inhibitors or scavengers and exogenous donors were dissolved in double distilled water and filtered through 0.22-μm diameter microporous membranes before use. The concentration of the above chemicals was according to our previous studies (Wang et al., 2011; Ren and Dai, 2012, 2013; Ren et al., 2013). Inhibitors or scavengers were sprayed on plant leaves and roots 1-day before the application of exogenous donors or AL12 inoculation. An equal volume of double distilled water was used as the control. The chemicals used in this study were chosen according to our previous studies. Unless stated otherwise, plants were harvested 15 days after the application of exogenous donors or AL12 inoculation. Each treatment was performed in triplicate.

### Ethylene extraction and measurement

Thirty-day-old plantlets treated with 5-mm AL12 mycelial disks or potato dextrose agar disks were harvested to determine the ethylene content at 0, 5, 10, 15, and 20 days. Fresh samples (1 g) were ground with 10 ml 0.1 M phosphate buffer solution (pH 7.4), and then centrifuged (3000 g, 20 min, 4°C). The supernatant was used for ethylene measurement. The production of ethylene was measured using the Plant ETH ELISA Kit (Shanghai Fankel Biological Technology Co., Ltd., China) following the manufacturer's instructions. At each time point, at least 15 plantlets were employed. Each treatment was performed in triplicate.

### Sesquiterpenoids extraction and gas chromatograph analysis

Harvested plants were dried at 30°C to a constant weight. After dry weights were measured, total sesquiterpenoids were extracted from whole plantlets according to Wang et al. (2015a). Briefly, dried plants were ground to a fine powder and one gram of plant powder was extracted with 4 mL cyclohexane for 10 h. After sonication (15 min, 60 Hz) and centrifugation (5000 g, 5 min, 4°C), total sesquiterpenoids extracts were dried over anhydrous sodium sulfate and filtered through 0.22-μm diameter microporous membranes, and then stored in dark glass bottles at 4°C before gas chromatography (GC) analysis.

GC analysis was conducted using an Agilent 7890A GC equipped with a fame ionization detector (Agilent, Santa Clara, CA, USA). GC operating conditions were as follows. An Agilent DB-1ms column (30 m × 0.32 mm × 0.10 μm) was used with the temperature program according to the method established in our laboratory (Zhou et al., 2015). The column temperature was held at 100°C for 4 min after injection, increased by 10°C min^−1^ to 140°C, held for 10 min, increased by 10°C min^−1^ to 220°C, held for 10 min, increased by 10°C min^−1^ to 260°C, and held for 2 min. High-purity nitrogen was used as a carrier at a flow rate of 0.8 mL min^−1^, and 1 μL was injected onto the column at injection temperature of 240°C. The detector temperature was set at 350°C and the pre-column pressure was 70 KPa. Seven sesquiterpenoids (including β-caryophyllene, zingiberene, β-sesquiphellandrene, caryophyllene oxide, hinesol, β-eudesmol, and atractylone) were identified according to the retention times of authentic standards (Ren and Dai, 2012; Wang et al., 2015a; Zhou et al., 2015), and their retention time (min) were 11.136, 12.999, 13.827, 15.549, 17.702, 17.946, and 18.535 respectively (Supplemental Figure S1). Standard curves were constructed for quantitative measurement of sesquiterpenoids. At each time point, at least 15 plantlets were employed. Each treatment was performed in triplicate.

### JA extraction and measurement

JA was extracted according to the established method (Engelberth et al., 2003; Ren and Dai, 2012). Five grams of plant materials were ground in liquid nitrogen and extracted with 20 mL H_2_O: acetone (30:70, v:v). Samples were stored in dark glass bottles at 4°C for analysis. JA content was measured using the Plant JA ELISA Kit (Shanghai Fankel Biological Technology Co., Ltd., China) following the manufacturer's instructions.

### SA extraction and HPLC analysis

SA was extracted according to our previous method with some modification (Wang et al., 2015a). Briefly, five grams of plant materials were ground in liquid nitrogen and extracted with 5 mL methanol for 10 min by sonication (60 Hz). After centrifugation (12,000 g, 5 min, 4°C), the supernatant was collected. The residue was extracted with 5 mL methanol for 10 min by sonication (60 Hz) and centrifuged (12,000 g, 5 min, 4°C) three times. The combined supernatant was mixed with 10 μL 0.2 M NaOH, evaporated under vacuum to dryness, dissolved in 250 μL of 5% trichloroacetic acid. The mixture was subjected to three consecutive liquid-liquid extractions with 800 μL ethyl acetate: cyclohexane (1:1, v/v). The combined organic phase was mixed with 60 μL 0.2 M acetate buffer (NaOAc) (pH 5.5), and evaporated under vacuum to dryness, dissolved in 600 μL mobile phase (methanol:2% acetic acid:H_2_O, 50:40:10, v:v:v), and filtered using a 0.22-μm diameter microporous membrane for analysis.

SA was quantified via high-performance liquid chromatography (HPLC) using an Agilent C18 column (250 × 4.6 mm, 5 μm) according to our previous method (Wang et al., 2011, 2015a; Ren and Dai, 2012). An Agilent 1290 Infinity (Agilent Technology, Germany) with UV detector and Agilent Chem-Station Software were used for quantification of SA. The injection volume was 20 μL and the column temperature was 25°C. The detection wavelength was set at 290 nm and isocratic elution was used at a flow rate of 0.5 mL min^−1^. Qualification and quantification analyses were based on comparison with SA standard. The SA peak in the fresh samples was identified by comparing retention time and area with that of the matching standard.

### Extraction and measurement of NO and H_2_O_2_

The production of NO and H_2_O_2_ was measured using the NO or H_2_O_2_ assay kit (Nanjing Jiancheng Bio-engineering Institute, China) following the manufacturer's instructions (Wang et al., 2011). Fresh sample of plant materials (1 g) were ground with 5 mL of 40 mM 4-(2-hydroxyethyl)-1-piperazineethanesulfonic acid (pH 7.2) for NO, or 5 mL of double distilled water for H_2_O_2_. After centrifugation (14,000 g, 10 min, 4°C), the supernatant was used for the measurement of NO and H_2_O_2_, respectively.

### Statistical analysis

Each experiment was performed in triplicate, and the whole experimental setup was repeated in triplicate with other batches of plant material to examine the reproducibility. The means and standard deviations (SD) were calculated using SPSS Statistics 17.0 software (SPSS Inc., Chicago, USA). The independent-Samples *T*-Test was used for statistical evaluation between two treatments. The one-way analysis of variance (ANOVA) followed by Tukey's multiple-comparison test (*P* < 0.05) was used for statistical evaluation between more than two treatments. The ANOVA was performed separately on sesquiterpenoids, ET, JA, SA, H_2_O_2_, and NO. Bars represent standard deviations. Asterisks denote significant differences from the control (*t*-test; ^*^p < 0.05; ^**^p < 0.01). Values followed by different types of lowercase letters (e.g., a, b, c; a′, b′, c′; a″, b″, c″) differ significantly at *P* = 0.05.

## Results

### Involvement of ethylene in AL12-induced sesquiterpenoids accumulation

**Figure 1:**
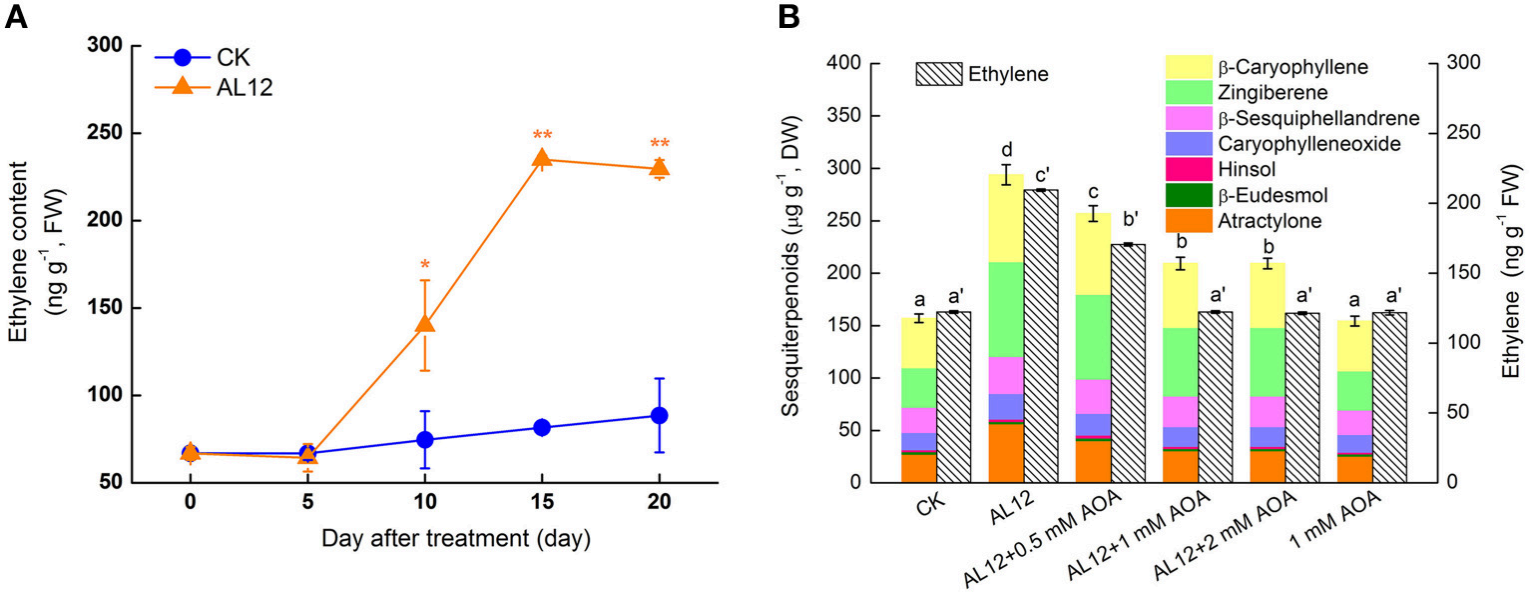
**Involvement of ethylene in endophytic fungus AL12-induced sesquiterpenoids accumulation in *Atractylodes lancea* plantlets**. **(A)** AL12-induced ethylene generation in plantlets. Thirty-day-old plantlets treated with 5-mm AL12 mycelial disks were harvested for ethylene measurement at 0, 5, 10, 15, and 20 day. Controls were established using equal sized potato dextrose agar disks. Values are the means of three independent experiments. Bars represent standard deviations. Asterisks denote significant differences from the control (*t*-test; ^*^P < 0.05; ^**^P < 0.01). **(B)** Effects of AOA (ethylene inhibitor) on AL12-induced sesquiterpenoids accumulation after 15 days. Inhibitors (0.5, 1, or 2 mM AOA) were added 1 day prior to AL12 inoculation. Controls were established using equal sized potato dextrose agar disks. Values are the means of three independent experiments ± SD. Bars with different lowercase letters (e.g., a, b, c; a′, b′, c′) are significantly different (*P* < 0.05).

The ET contents of *A. lancea* increased significantly after endophytic fungus AL12 inoculation (Figure 1A), indicating that AL12 may trigger the biosynthesis of ET in *A. lancea*. AOA, an inhibitor of ACC biosynthesis, is usually applied as an inhibiter of ethylene production. To investigate whether ET was involved in AL12-induced sesquiterpenoids accumulation, the effects of AOA on the production of AL12-induced sesquiterpenoids in *A. lancea* were determined.

As shown in Figure 1B, AOA suppressed not only AL12-triggered ET production, but also AL12-induced sesquiterpenoids accumulation. The sesquiterpenoids concentrations of plantlets treated with 0.5, 1, and 2 mM AOA were 12.69, 28.7%, and 28.77% lower than that of AL12-innoculated plantlets, respectively. The results suggested that the production of ET is involved in AL12-induced sesquiterpenoids accumulation of *A. lancea*. And, 1 mM AOA was chosen for the following experiments.

### Dependence of ethylene-induced sesquiterpenoids accumulation on JA

**Figure 2:**
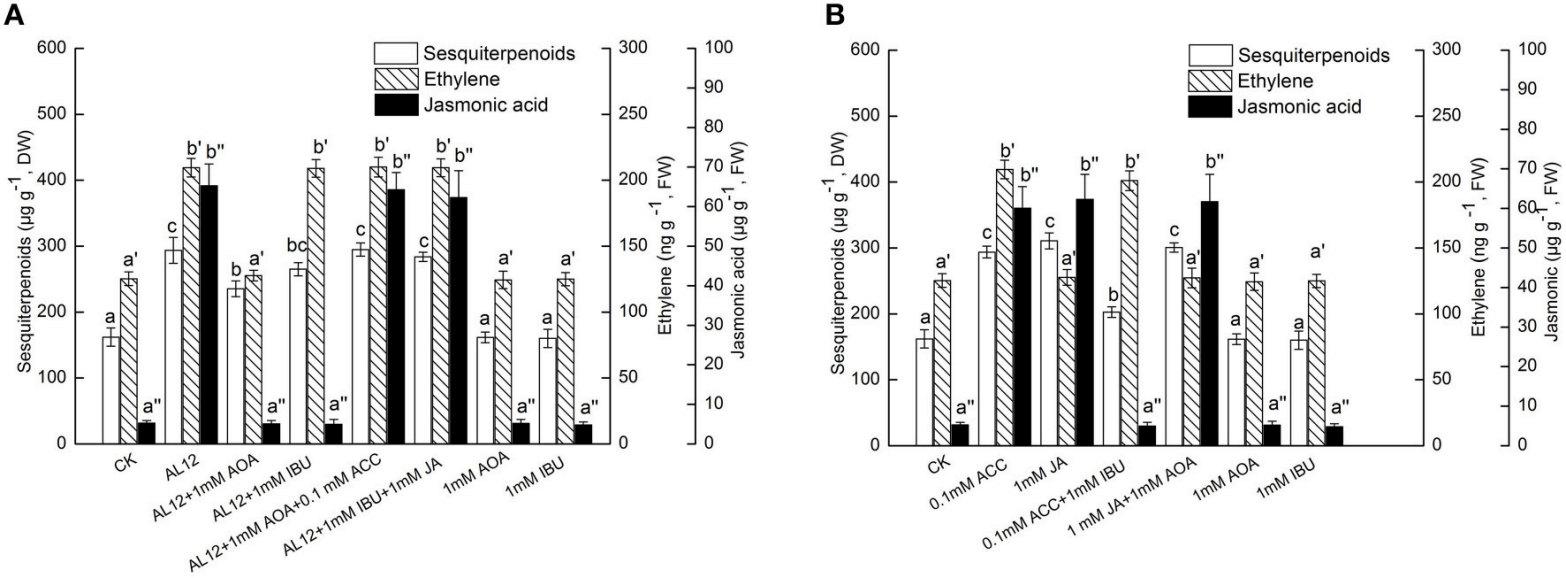
**(A)** Interaction between ethylene and JA signaling pathways for endophytic fungus AL12-induced sesquiterpenoids accumulation in *Atractylodes lancea* plantlets. Thirty-day-old plantlets treated with 5-mm AL12 mycelial disks, 1 mM AOA, 1 mM IBU, 0.1 mM ACC, and 1 mM JA were harvested after 15 days to determine the sesquiterpenoids, ethylene and JA contents. Inhibitors were added 1 day prior to endophytic fungus AL12 inoculation or exogenous signal donor application. Controls were established using equal sized potato dextrose agar disks. Values are the means of three independent experiments ± SD. Bars with different lowercase letters (e.g., a, b, c; a′, b′, c′; a″, b″, c″) are significantly different (*P* < 0.05). **(B)** Dependence of ethylene-induced sesquiterpenoids accumulation on JA in *Atractylodes lancea* plantlets. Thirty-day-old plantlets treated with 0.1 mM ACC, 1 mM JA, 1 mM AOA, and 1 mM IBU were harvested 15 days later to determine the sesquiterpenoids, ethylene and JA contents. Inhibitors were added 1 day prior to exogenous signal donor application. Controls were established using equal volume of double distilled water. Values are the means of three independent experiments ± SD. Bars with different lowercase letters (e.g., a, b, c; a′, b′, c′; a″, b″, c″) are significantly different (*P* < 0.05).

IBU is an inhibitor of the octadecanoid pathway that synthesizes JA, and is applied as a specific inhibitor of JA (Ren and Dai, 2012). Figure 2A showed that both AOA and IBU could significantly suppress AL12-induced sesquiterpenoids accumulation in *A. lancea*. AOA can strongly suppress AL12-induced JA generation; whereas IBU did not affect AL12-triggered ET generation (Figure 2A). This result suggested that the ET and JA signaling pathway were connected, and that ET might act as an upstream signal of AL12-induced JA generation and sesquiterpenoids production in the *A. lancea* plantlets. The data showing that the application of exogenous ACC and JA could reverse the suppression of AL12-induced sesquiterpenoids production by AOA and IBU further confirmed our results (Figure 2A).

To investigate if exogenous ET-induced sesquiterpenoids accumulation also depended on JA signaling, the effects of exogenous ACC, JA, AOA, and IBU on the accumulation of sesquiterpenoids, ET and JA were determined. Figure 2B showed that both exogenous ACC and JA could significantly induce sesquiterpenoids accumulation in *A. lancea*. The increased sesquiterpenoids accumulation triggered by ACC was partly suppressed by IBU; whereas the increased sesquiterpenoids accumulation triggered by JA was not affected by AOA (Figure 2B). In addition, exogenous ACC could trigger JA generation; whereas exogenous JA did not affect ET production (Figure 2B). And, exogenous AOA or IBU itself had no adverse effect on the accumulation of sesquiterpenoids, ET and JA in the *A. lancea* plantlets compared to the control (Figure 2B). These results further demonstrated that ET acted as an upstream signal of JA in AL12-induced sesquiterpenoids accumulation of *A. lancea*. Additionally, IBU could not completely abolish ACC-induced sesquiterpenoids accumulation (Figure 2B), suggesting that ET-induced sesquiterpenoids accumulation is not solely dependent on the JA signaling pathway.

### Dependence of ethylene-induced sesquiterpenoids accumulation on SA

**Figure 3:**
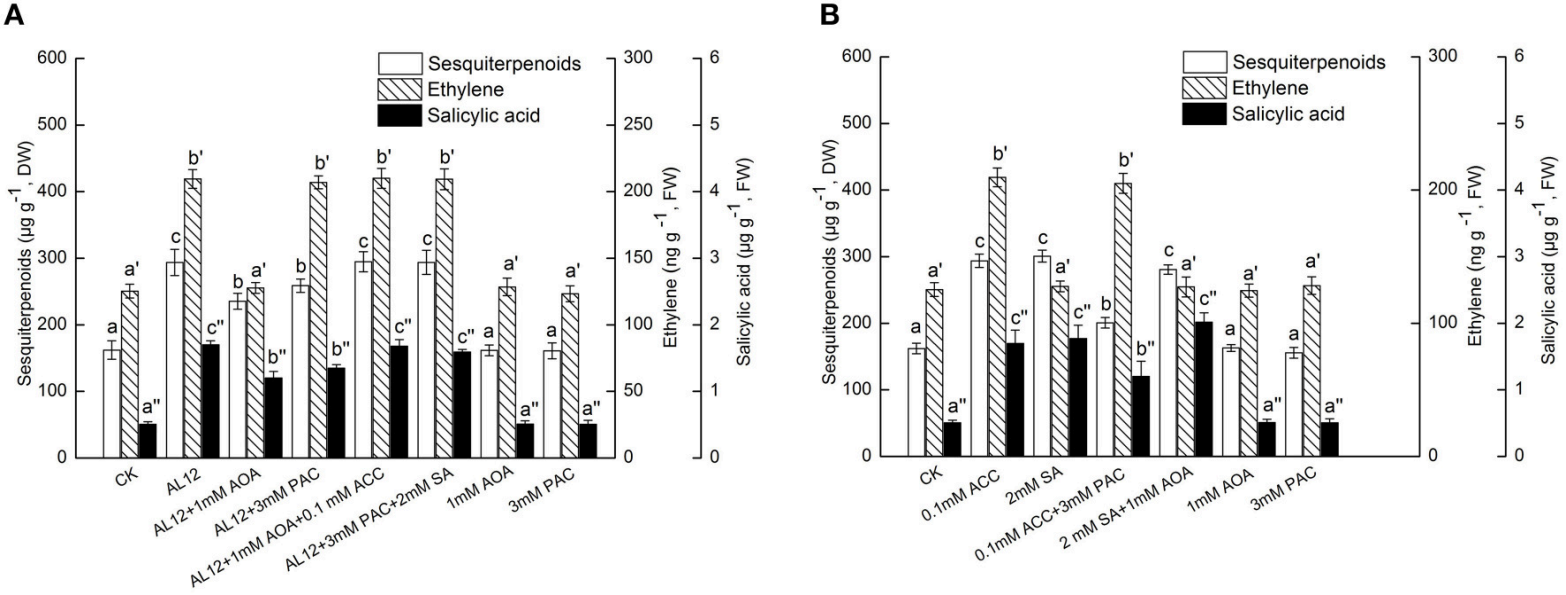
**(A)** Interaction between ethylene and SA signaling pathways for endophytic fungus AL12-induced sesquiterpenoids accumulation in *Atractylodes lancea* plantlets. Thirty-day-old plantlets treated with 5-mm AL12 mycelial disks, 1 mM AOA, 3 mM PAC, 0.1 mM ACC, and 2 mM SA were harvested after 15 days to determine the sesquiterpenoids, ethylene and SA contents. Inhibitors were added 1 day prior to endophytic fungus AL12 inoculation or exogenous signal donor application. Controls were established using equal sized potato dextrose agar disks. Values are the means of three independent experiments ± SD. Bars with different lowercase letters (e.g., a, b, c; a′, b′, c′; a″, b″, c″) are significantly different (*P* < 0.05). **(B)** Dependence of ethylene-induced sesquiterpenoids accumulation on SA in *Atractylodes lancea* plantlets. Thirty-day-old plantlets treated with 0.1 mM ACC, 2 mM SA, 1 mM AOA, and 3 mM PAC were harvested 15 days later to determine the sesquiterpenoids, ethylene and SA contents. Inhibitors were added 1 day prior to exogenous signal donor application. Controls were established using equal volume of double distilled water. Values are the means of three independent experiments ± SD. Bars with different lowercase letters (e.g., a, b, c; a′, b′, c′; a″, b″, c″) are significantly different (*P* < 0.05).

PAC is an inhibitor of benzoic acid hydroxylase that related to SA biosynthesis (Wang et al., 2011; Ren and Dai, 2012), and is applied as SA-inhibitor in many plants. Figure 3A showed that both AOA and PAC could significantly suppress AL12-induced sesquiterpenoids accumulation in *A. lancea*. AOA can strongly suppress AL12-induced SA generation; whereas PAC did not affect AL12-triggered ET generation (Figure 3A). This result suggested that the ET and SA signaling pathway were connected, and that ET might act as an upstream signal of AL12-induced SA generation and sesquiterpenoids production in the *A. lancea* plantlets. The data showing that the application of exogenous ACC and SA could reverse the suppression of AL12-induced sesquiterpenoids production by AOA and PAC further confirmed our results (Figure 3A).

To investigate if exogenous ET-induced sesquiterpenoids accumulation also depended on SA signaling, the effects of exogenous ACC, SA, AOA, and PAC on the accumulation of sesquiterpenoids, ET and SA were determined. Figure 3B showed that both exogenous ACC and SA could significantly induce sesquiterpenoids accumulation in *A. lancea*. The increased sesquiterpenoids accumulation triggered by ACC was partly suppressed by PAC; whereas the increased sesquiterpenoids accumulation triggered by SA was not affected by AOA (Figure 3B). In addition, exogenous ACC could trigger SA generation; whereas exogenous SA did not affect ET production (Figure 3B). And, exogenous AOA or PAC itself had no adverse effect on the accumulation of sesquiterpenoids, ET and SA in the *A. lancea* plantlets compared to the control (Figure 3B). These results further demonstrated that ET acted as an upstream signal of SA in AL12-induced sesquiterpenoids accumulation of *A. lancea*. Additionally, PAC could not completely abolish ACC-induced sesquiterpenoids accumulation (Figure 3B), suggesting that ET-induced sesquiterpenoids accumulation is not solely dependent on the SA signaling pathway.

### Ethylene acts as a downstream signal of H_2_O_2_

**Figure 4:**
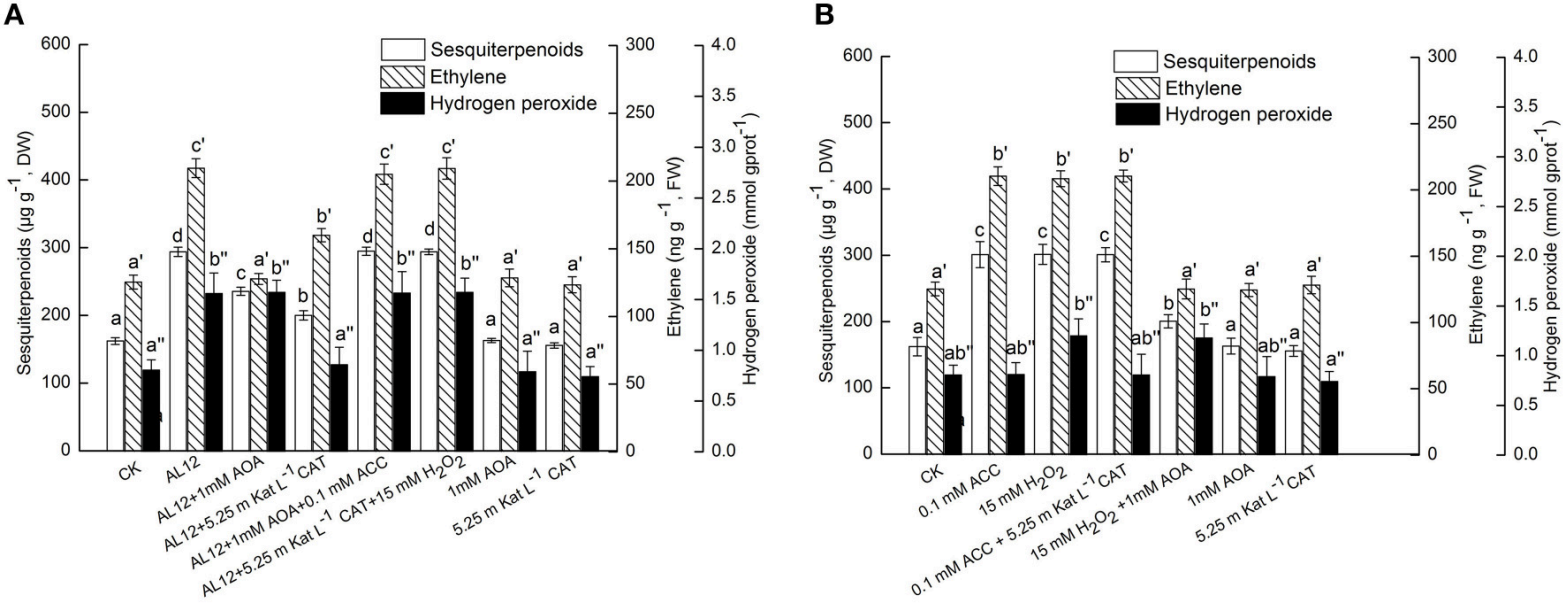
**(A)** Interaction between ethylene and H_2_O_2_ signaling pathways for endophytic fungus AL12-induced sesquiterpenoids accumulation in *Atractylodes lancea* plantlets. Thirty-day-old plantlets treated with 5-mm AL12 mycelial disks, 1 mM AOA, 5.25 m Kat L^−1^ CAT, 0.1 mM ACC and 15 mM H_2_O_2_ were harvested after 15 days to determine the sesquiterpenoids, ethylene and H_2_O_2_ contents. Inhibitors were added 1 day prior to endophytic fungus AL12 inoculation or exogenous signal donor application. Controls were established using equal sized potato dextrose agar disks. Values are the means of three independent experiments ± SD. Bars with different lowercase letters (e.g., a, b, c; a′, b′, c′; a″, b″, c″) are significantly different (*P* < 0.05). **(B)** Dependence of H_2_O_2_-induced sesquiterpenoids accumulation on ethylene in *Atractylodes lancea* plantlets. Thirty-day-old plantlets treated with 0.1 mM ACC, 15 mM H_2_O_2_, 1 mM AOA, and 5.25 m Kat L^−1^ CAT were harvested 15 days later to determine the sesquiterpenoids, ethylene and H_2_O_2_ contents. Inhibitors were added 1 day prior to exogenous signal donor application. Controls were established using equal volume of double distilled water. Values are the means of three independent experiments ± SD. Bars with different lowercase letters (e.g., a, b, c; a′, b′, c′; a″, b″, c″) are significantly different (*P* < 0.05).

CAT is an inhibitor of NADPH oxidase, and is applied as H_2_O_2_ scavenger (Wang et al., 2011; Ren and Dai, 2012). Figure 4A showed that both AOA and CAT could significantly suppress AL12-induced sesquiterpenoids accumulation in *A. lancea*. CAT can strongly suppress AL12-induced ET generation; whereas AOA did not affect AL12-triggered H_2_O_2_ generation (Figure 4A). This result suggested that the ET and H_2_O_2_ signaling pathway were connected, and that ET might act as a downstream signal in H_2_O_2_-mediated sesquiterpenoids accumulation induced by AL12. The data showing that the application of exogenous ACC and H_2_O_2_ could reverse the suppression of AL12-induced sesquiterpenoids production by AOA and CAT further confirmed our results (Figure 4A).

To investigate if exogenous ET also acted as a downstream signal in H_2_O_2_-mediated sesquiterpenoids accumulation, the effects of exogenous ACC, H_2_O_2_, AOA, and CAT on the accumulation of sesquiterpenoids, ET and H_2_O_2_ were determined. Figure 4B showed that both exogenous ACC and H_2_O_2_ could significantly induce sesquiterpenoids accumulation in *A. lancea*. The increased sesquiterpenoids accumulation triggered by H_2_O_2_ was partly suppressed by AOA; whereas the increased sesquiterpenoids accumulation triggered by ACC was not affected by CAT (Figure 4B). In addition, exogenous H_2_O_2_ could trigger ET generation; whereas exogenous ACC did not affect H_2_O_2_ production (Figure 4B). And, exogenous AOA or CAT itself had no adverse effect on the accumulation of sesquiterpenoids, ET and H_2_O_2_ in the *A. lancea* plantlets compared to the control (Figure 4B). These results further demonstrated that ET acted as a downstream signal in H_2_O_2_-mediated sesquiterpenoids accumulation induced by AL12. And, AOA could not completely abolish H_2_O_2_-induced sesquiterpenoids accumulation (Figure 4B), suggesting that H_2_O_2_ was not the sole upstream signal of ET.

### Ethylene acts as a downstream signal of NO

**Figure 5:**
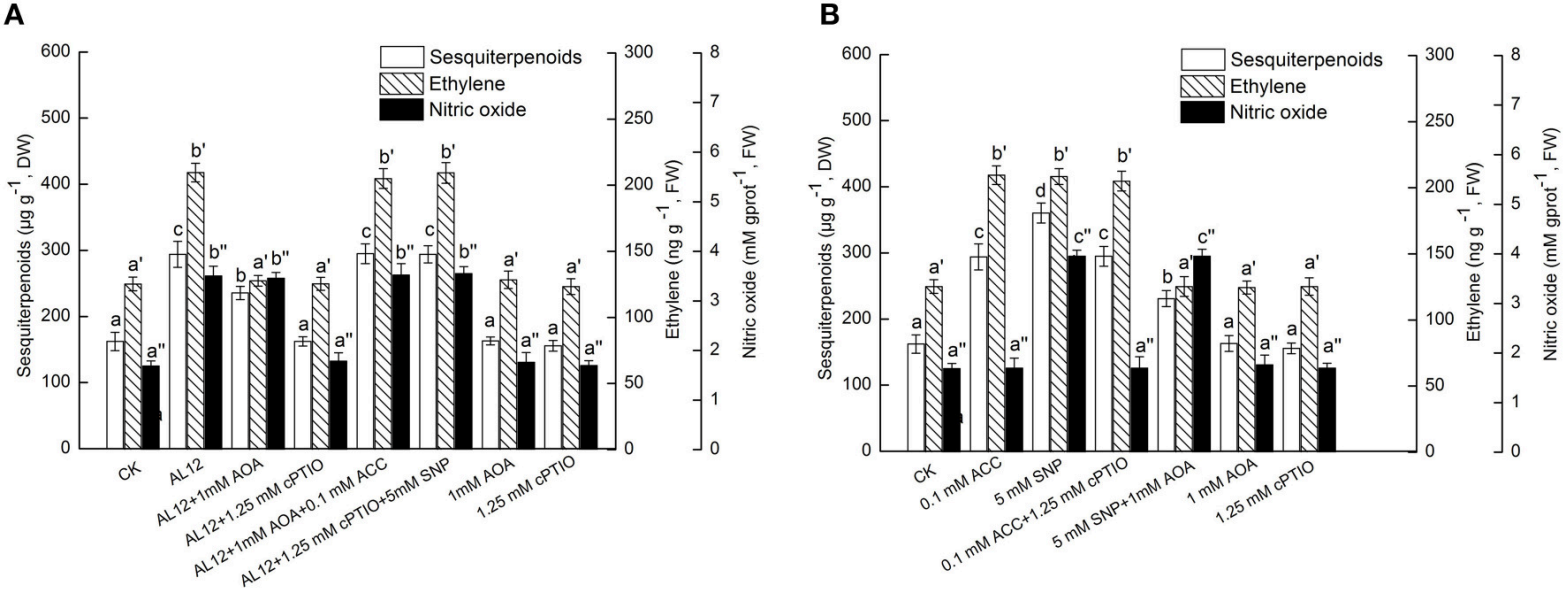
**(A)** Interaction between ethylene and NO signaling pathways for endophytic fungus AL12-induced sesquiterpenoids accumulation in *Atractylodes lancea* plantlets. Thirty-day-old plantlets treated with 5-mm AL12 mycelial disks, 1 mM AOA, 1.25 mM cPTIO, 0.1 mM ACC, and 5 mM SNP were harvested after 15 days to determine the sesquiterpenoids, ethylene and NO contents. Inhibitors were added 1 day prior to endophytic fungus AL12 inoculation or exogenous signal donor application. Controls were established using equal sized potato dextrose agar disks. Values are the means of three independent experiments ± SD. Bars with different lowercase letters (e.g., a, b, c; a′, b′, c′; a″, b″, c″) are significantly different (*P* < 0.05). **(B)** Dependence of NO-induced sesquiterpenoids accumulation on ethylene in *Atractylodes lancea* plantlets. Thirty-day-old plantlets treated with 0.1 mM ACC, 5 mM SNP, 1 mM AOA, and 1.25 mM cPTIO were harvested 15 days later to determine the sesquiterpenoids, ethylene and NO contents. Inhibitors were added 1 day prior to exogenous signal donor application. Controls were established using equal volume of double distilled water. Values are the means of three independent experiments ± SD. Bars with different lowercase letters (e.g., a, b, c; a′, b′, c′; a″, b″, c″) are significantly different (*P* < 0.05).

NO specific scavenge cPTIO and exogenous NO donor SNP were applied in this work. Figure 5A showed that both AOA and cPTIO could significantly suppress AL12-induced sesquiterpenoids accumulation in *A. lancea*. NO specific scavenge cPTIO can strongly suppress AL12-induced ET generation; whereas AOA did not affect AL12-triggered NO generation (Figure 5A). This result suggested that the ET and NO signaling pathway were connected, and that ET might act as a downstream signal in NO-mediated sesquiterpenoids accumulation induced by AL12. The data showing that the application of exogenous ACC and SNP could reverse the suppression of AL12-induced sesquiterpenoids production by AOA and cPTIO further confirmed our results (Figure 5A).

To investigate if exogenous ET also acted as a downstream signal in NO-mediated sesquiterpenoids accumulation, the effects of exogenous ACC, SNP, AOA, and cPTIO on the accumulation of sesquiterpenoids, ET and NO were determined. Figure 5B showed that both exogenous ACC and SNP could significantly induce sesquiterpenoids accumulation in *A. lancea*. The increased sesquiterpenoids accumulation triggered by SNP was partly suppressed by AOA; whereas the increased sesquiterpenoids accumulation triggered by ACC was not affected by cPTIO (Figure 5B). In addition, exogenous SNP could trigger ET generation; whereas exogenous ACC did not affect NO production (Figure 5B). And, exogenous AOA or cPTIO itself had no adverse effect on the accumulation of sesquiterpenoids, ET and NO in the *A. lancea* plantlets compared to the control (Figure 5B). These results further demonstrated that ET acted as a downstream signal in NO-mediated sesquiterpenoids accumulation induced by AL12. And, AOA could not completely abolish SNP-induced sesquiterpenoids accumulation (Figure 5B), suggesting that NO was not the sole upstream signal of ET.

### Contributions of five signals in AL12-induced sesquiterpenoids accumulation

**Figure 6:**
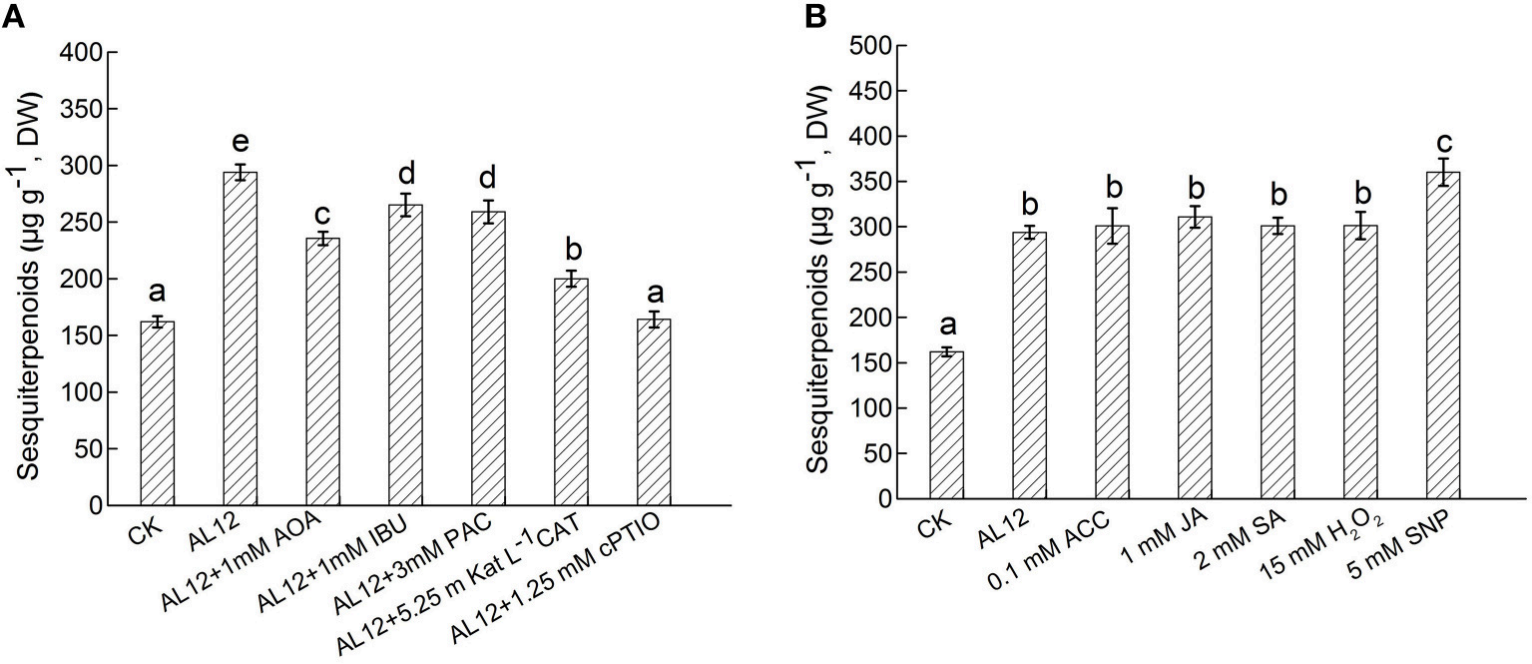
**(A)** Contributions of five signals in endophytic fungus AL12-induced sesquiterpenoids accumulation in *Atractylodes lancea* plantlets. Thirty-day-old plantlets treated with 5-mm AL12 mycelial disks, 1 mM AOA, 1 mM IBU, 3 mM PAC, 5.25 m Kat L^−1^ CAT and 1.25 mM cPTIO were harvested after 15 days to determine the sesquiterpenoids content. Inhibitors were added 1 day prior to endophytic fungus AL12 inoculation. Controls were established using equal sized potato dextrose agar disks. Values are the means of three independent experiments ± SD. Bars with different lowercase letters (e.g., a, b, c) are significantly different (*P* < 0.05). **(B)** Comparison of five exogenous signal donor. Thirty-day-old plantlets treated with 5-mm AL12 mycelial disks, 0.1 mM ACC, 1 mM JA, 2 mM SA, 15 mM H_2_O_2_, and 5 mM SNP were harvested after 15 days to determine the sesquiterpenoids content. Controls were established using equal volume of double distilled water. Values are the means of three independent experiments ± SD. Bars with different lowercase letters (e.g., a, b, c) are significantly different (*P* < 0.05).

The endophytic fungus AL12 induced sesquiterpenoids accumulation of the *A. lancea* plantlets through multiple signals. Here, we compared the contributions of ET, JA, SA, H_2_O_2_, and NO signals in AL12-induced sesquiterpenoids in *A. lancea*. As shown by Figure 6A, AOA, IBU, PAC, CAT, and cPTIO all significantly suppressed AL12-induced sesquiterpenoids accumulation. And the inhibition effect of cPTIO, CAT, and AOA on sesquiterpenoids accumulation were stronger than other inhibitors (Figure 6A), indicating that ET, H_2_O_2_, and NO signaling acted as three main pathways in AL12-induced sesquiterpenoids accumulation. Further, we compared the contributions of exogenous ACC, JA, SA, H_2_O_2_, and SNP on sesquiterpenoids accumulation. As shown by Figure 6B, exogenous ACC, JA, SA, H_2_O_2_, and SNP significantly induced sesquiterpenoids accumulation, and the induction effect of SNP was strongest.

### Comparison of signaling pathways involved in sesquiterpenoids biosynthesis induced by different endophytes

**Figure 7:**
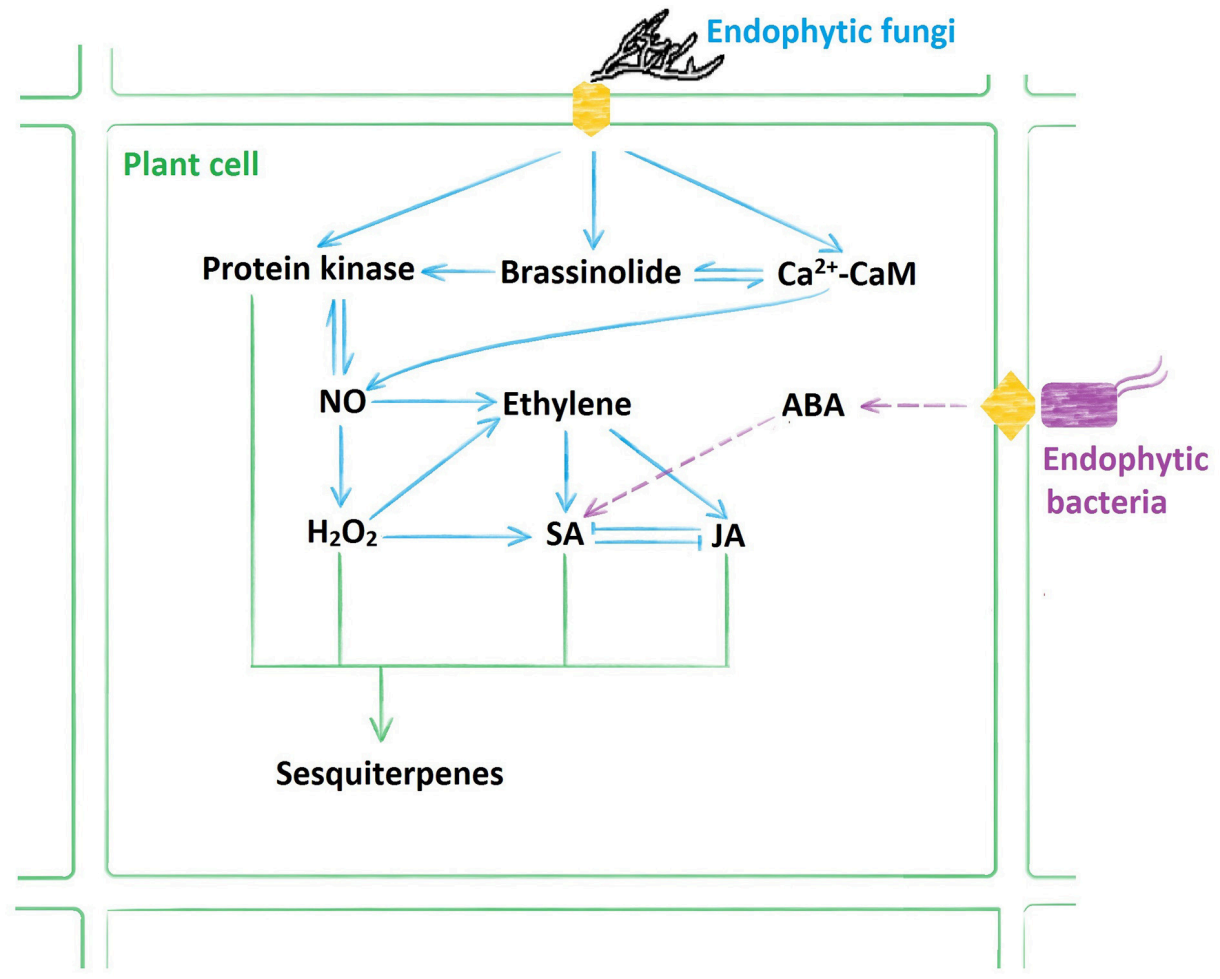
**Model illustrating multiple signaling pathways involved in sesquiterpenoids biosynthesis induced by the endophytes**. The endophytic fungus AL12 induced sesquiterpenoids biosynthesis through NO, H_2_O_2_, ET, SA, JA, brassinosteroid (BR), and calcium (Ca^2+^) signaling pathways; whereas the endophytic bacteria ALEB16 induced sesquiterpenoids biosynthesis through abscisic acid (ABA) and SA signaling pathways.

Based on our previous results, we summarized the signaling pathways induced by the endophytic fungi and the endophytic bacteria (Wang et al., 2011, 2015a; Ren and Dai, 2012, 2013; Ren et al., 2013). As shown by Figure 7, the endophytic fungus AL12 induced sesquiterpenoids biosynthesis through NO, H_2_O_2_, ET, SA, JA, brassinosteroid (BR), and calcium (Ca^2+^) signals; whereas the endophytic bacteria ALEB16 induced sesquiterpenoids biosynthesis through abscisic acid (ABA) and SA signals. Therefore, sesquiterpenoids biosynthesis of *A. lancea* triggered by different endophytes may not be the same.

## Discussion

Plant secondary metabolites plays an important role in plant defense system, and the accumulation of active compounds is regulated by cross-talking signaling cascades (Jacobo-Velázquez et al., 2015). This study showed that ET was involved in the signaling pathway for AL12-induced sesquiterpenoids accumulation of the *A. lancea* plantlets (Figure 1). Some reports indicated that ET may be a common signal that can be used to induce the biosynthesis of diverse secondary metabolites (Zhao et al., 2004; Arimura et al., 2007; Liu et al., 2012; Rahimi et al., 2015). As shown by Figures 6A,B, ET and NO signaling acted as two main pathways in AL12-induced sesquiterpenoids accumulation. Ethephon, an agricultural plant ripening agent, can produce ethylene when dissolves in water, and is very cheap and easy to purchase (Navet et al., 2003; Ban et al., 2007). Ethephon may be used as a signal in inducing the biosynthesis of secondary metabolites in medicinal plants. The application of ethephon will promote the development of medicinal plants industry, and will help to solve the problems of medicinal plants quality.

Different signaling pathways act synergistically or antagonistically, providing a powerful regulatory system for plants adapting to various environmental signals (Verhage et al., 2010). Intriguingly, ET interacts with more than one signal in plants. Typically, JA/ET signaling mediates plants resistance against necrotrophic pathogens, and JA and ET signaling interacts synergistically or antagonistically (Verhage et al., 2010). It has been reported that ET and JA signaling interacted in yeast elicitor induction of β-thujaplicin, with JA signaling acting as a main control and ET as a fine modulator (Zhao et al., 2004). Another study shows that ET and JA played a positive role in the biosynthesis of a tetraterpenoid, lycopene, in tomato fruits, with JA functioning independently of ET (Liu et al., 2012). In this study, we investigate the possible relationship between ET and JA signaling. As suggested by Figure 2, ET acted as the sole upstream signal of AL12-induced JA generation and sesquiterpenoids production in the *A. lancea* plantlets. Our previous study has shown that JA has a complementary interaction with the SA signaling pathway in AL12 induction of sesquiterpenoids (Ren and Dai, 2012). Therefore, we investigate the possible relationship between ET and SA signaling in this study. As indicated by Figure 3, ET acted as an upstream signal of AL12-induced SA generation and sesquiterpenoids production in the *A. lancea* plantlets. Therefore, ET, JA, and SA signaling interacted in AL12 induction of sesquiterpenoids accumulation, with ET is the upstream signal of JA and SA (Figures 2, 3), and JA has a complementary interaction with the SA signaling (Ren and Dai, 2012). It has been reviewed that ET, JA, and, SA are three endogenous plant signaling molecules involved in plant immunity (Verhage et al., 2010). It has been reported that volatiles compounds from the rhizobacterium *Bacillus subtilis* GB03 up-regulate several genes related to ET biosynthesis and response, JA response, and SA response in *Arabidopsis* (Kwon et al., 2010). Here, we speculate that the SA, JA, and ET signaling pathways maybe utilized and/or modulated in different ways in different plant species.

ET interacts with other signals such as NO and H_2_O_2_. It has been reported that wound-induced accumulation of plant secondary metabolites is mediated by reactive oxygen species (ROS), ET and JA, whereas ET and JA are essential to modulate reactive oxygen species ROS levels (Jacobo-Velázquez et al., 2015). Our previous studies have revealed that NO acted as an upstream signal of H_2_O_2_ and SA, H_2_O_2_ regulated SA production (Wang et al., 2011), and JA acted as a downstream signal of NO and H_2_O_2_ (Ren and Dai, 2012). As shown by Figures 2, 3, ET is the upstream signal of JA and SA. In this study, we investigate the possible relationship between ET and H_2_O_2_ or NO signaling. As indicated by Figures 4, 5, ET acted as a downstream signal in NO- and H_2_O_2_-mediated sesquiterpenoids accumulation induced by AL12. To summarize, ET is the upstream signal of JA and SA (Figures 2, 3), and the downstream signal of NO and H_2_O_2_ (Figures 4, 5), showing that ET acted as an important signal mediating AL12-induced sesquiterpenoids accumulation.

Multiple signaling pathways connected and mediated plant defense-related genes expression, and also induced secondary metabolites biosynthesis. As shown by Figure 7, signaling pathways induced by the endophytic fungi and the endophytic bacteria mediating sesquiterpenoids biosynthesis of *A. lancea* are of some differences. It has reported that *Arabidopsis thaliana* MYC2, a basic helix-loop-helix transcription factor, could directly bind to promoters of the sesquiterpene synthase genes *TPS21* and *TPS11*, thus activating their expression (Hong et al., 2012). Exogenous gibberellin and JA could induce MYC2, thus activating the expression of *TPS21* and *TPS11* (Hong et al., 2012). The activation of *TPS21* and *TPS11* induced the emission of sesquiterpene, especially (E)-β-caryophyllene (Hong et al., 2012). Since multiple signaling pathways were induced by the endophytic fungus, is there one or one class of common targets? And, proteomics, transcriptomics, and other advanced technologies are required to be employed to study the cross-talk between multiple signaling pathways, which will help to understand their roles in promoting the biosynthesis of the secondary metabolites.

Cultivated plantlets of *A. lancea* are of relatively low survival ratio and sesquiterpenoids content (Zhou et al., 2014, 2015). Our previous studies showed that AL12 can promote plant growth, induce secondary metabolites accumulation, and enhance plant defense responses in the plantlets of *A. lancea* (Wang et al., 2012). Several studies have reported that ethylene could increase cell expansion, stimulate internode expansion, improve seed germination, stimulate fruit ripening, and help plants resisting against various stresses such as *Pseudomonas syringae*, high salt, heavy metal, flooding, and drought (Arc et al., 2013; Steffens, 2014; Arraes et al., 2015; Bakshi et al., 2015; Guan et al., 2015; Wei et al., 2015). It has been reviewed that, ethylene, JA, and SA are three key signals mediating plant defense against microbial attack (Kunkel and Brooks, 2002). Therefore, we speculate that ethylene, JA, SA and other signals induced by the endophytic fungus AL12 help *A. lancea* resisting adverse environmental factors, promote plant growth, and also induce secondary metabolites biosynthesis. It also indicated that endophytes are of potential application value in cultivating of medicinal plants.

Many medicinal plants contain active secondary metabolites (such as terpenes, flavones, and alkaloids), and are an important source of modern drugs (Wangchuk and Tobgay, 2015). You-You Tu, the mother of artemisinin, was awarded Nobel Prize in medicine in 2015. The development of traditional medicinal plants gradually becomes a hot issue, and endophytes-medicinal plants interactions will receive much attention. Sesquiterpenoids are the main medicinal compounds in *A. lancea* (Wang et al., 2008). Several endophytes, such as *Gilmaniella* sp. AL12, *Acinetobacter* sp. ALEB16, and ALEB7B, can establish symbiotic relationships with *A. lancea*, and also greatly promote sesquiterpenoids accumulation in the herb (Wang et al., 2012, 2015a; Zhou et al., 2015), indicating that these endophytes are of potential application value in guaranteeing the quality of medicinal materials. In recent years, *A. lancea*-endophytes interactions gradually become one model of medicinal plants-endophytes interactions (Wang et al., 2011, 2012, 2015a,b; Ren and Dai, 2012, 2013; Ren et al., 2013; Yang et al., 2013, 2014; Zhou et al., 2014, 2015), and provides a theoretical reference for the biosynthesis of other medicinal compounds. In this work, plant materials of *A. lancea* obtained from plant tissue culture were sterile, and were appropriate for investigating the effect of one specific factor on plant materials without the distraction of other factors. Plant tissue culture will help to study the effects of endophytes on plants, thus helping to understand plant-endophyte interactions.

## Conclusions

In summary, this study showed that ethylene is an upstream signal of JA and SA, and a downstream signal of NO and H_2_O_2_, and acts as an important signal mediating AL12-induced sesquiterpenoids accumulation. And, signaling pathways induced by the endophytic fungi and the endophytic bacteria mediating sesquiterpenoids biosynthesis of *A. lancea* are of some differences. This study comprehensively demonstrates the signaling pathways of sesquiterpenoids biosynthesis, and provides a theoretical basis for the industrialization of active compounds in *A. lancea*.

## Author contributions

JY designed the experiments, carried out most of the experimental work, analyzed data, and wrote the manuscript. KS and MY helped for the measurement of ET, NO, and H_2_O_2_. CC supervised the work. All authors read and approved the final manuscript.

### Conflict of interest statement

The authors declare that the research was conducted in the absence of any commercial or financial relationships that could be construed as a potential conflict of interest.

## Supplementary material

Supplementary Figure S1 **Gas chromatograph analysis of sesquiterpenoids in *Atractylodes lancea***.

## Abbreviations

A. lancea: Atractylodes lancea
AL12: Endophytic fungus *Gilmaniella* sp. AL12
NO: Nitric oxide
H_2_O_2_: Hydrogen peroxide
SA: Salicylic acid
JA: Jasmonic acid
ET: Ethylene
AOA: Aminooxyacetic acid
IBU: Ibuprofen
PAC: Paclobutrazol
CAT: Catalase
cPTIO: 2-(4-Carboxyphenyl)-4,4,5,5-tetramethylimidazoline-1-oxyl-3-oxide potassium salt
ACC: 1-aminocyclopropane-1-carboxylic acid
SNP: Sodium nitroprusside
GC: Gas chromatography
HPLC: High-performance liquid chromatography.

## References

[B1] Arc E. Sechet J. Corbineau F. Rajjou L. Marion-Poll A. (2013). ABA crosstalk with ethylene and nitric oxide in seed dormancy and germination. Front. Plant Sci. 4:63. 10.3389/fpls.2013.00063 23531630 PMC3607800

[B2] Arimura G.-I. Garms S. Maffei M. Bossi S. Schulze B. Leitner M.. (2007). Herbivore-induced terpenoid emission in *Medicago truncatula*: concerted action of jasmonate, ethylene and calcium signaling. Planta 227, 453–464. 10.1007/s00425-007-0631-y 17924138 PMC2756395

[B3] Arraes F. B. Beneventi M. A. Lisei de Sa M. E. Paixao J. F. Albuquerque E. V. Marin S. R.. (2015). Implications of ethylene biosynthesis and signaling in soybean drought stress tolerance. BMC Plant Biol. 15:213. 10.1186/s12870-015-0597-z 26335593 PMC4557918

[B4] Bakshi A. Shemansky J. M. Chang C. Binder B. M. (2015). History of research on the plant hormone ethylene. J. Plant Growth Regul. 34, 809–827. 10.1007/s00344-015-9522-9

[B5] Ban T. Kugishima M. Ogata T. Shiozaki S. Horiuchi S. Ueda H. (2007). Effect of ethephon (2-chloroethylphosphonic acid) on the fruit ripening characters of rabbiteye blueberry. Sci. Hortic. 112, 278–281. 10.1016/j.scienta.2006.12.027

[B6] Engelberth J. Schmelz E. A. Alborn H. T. Cardoza Y. J. Huang J. Tumlinson J. H. (2003). Simultaneous quantification of jasmonic acid and salicylic acid in plants by vapor-phase extraction and gas chromatography-chemical ionization-mass spectrometry. Anal. Biochem. 312, 242–250. 10.1016/S0003-2697(02)00466-9 12531212

[B7] Guan R. Su J. Meng X. Li S. Liu Y. Xu J.. (2015). Multilayered regulation of ethylene induction plays a positive role in arabidopsis resistance against *Pseudomonas syringae*. Plant Physiol. 169, 299–312. 10.1104/pp.15.00659 26265775 PMC4577408

[B8] Hong G. J. Xue X. Y. Mao Y. B. Wang L. J. Chen X. Y. (2012). Arabidopsis MYC2 interacts with DELLA proteins in regulating sesquiterpene synthase gene expression. Plant Cell 24, 2635–2648. 10.1105/tpc.112.098749 22669881 PMC3406894

[B9] Jacobo-Velázquez D. A. González-Agüero M. Cisneros-Zevallos L. (2015). Cross-talk between signaling pathways: the link between plant secondary metabolite production and wounding stress response. Sci. Rep. 5:8608. 10.1038/srep08608 25712739 PMC5390084

[B10] Ji L. Ao P. Pan J. G. Yang J. Y. Yang J. Hu S. L. (2001). [GC-MS analysis of essential oils from rhizomes of *Atractylodes lancea* (Thunb.) DC. and A. chinensis (DC.) Koidz]. Zhongguo Zhong Yao Za Zhi 26, 182–185. 12525038

[B11] Kunkel B. N. Brooks D. M. (2002). Cross talk between signaling pathways in pathogen defense. Curr. Opin. Plant Biol. 5, 325–331. 10.1016/S1369-5266(02)00275-3 12179966

[B12] Kwon Y. S. Ryu C. M. Lee S. Park H. B. Han K. S. Lee J. H.. (2010). Proteome analysis of Arabidopsis seedlings exposed to bacterial volatiles. Planta 232, 1355–1370. 10.1007/s00425-010-1259-x 20820802

[B13] Liu L. Wei J. Zhang M. Zhang L. Li C. Wang Q. (2012). Ethylene independent induction of lycopene biosynthesis in tomato fruits by jasmonates. J. Exp. Bot. 63, 5751–5761. 10.1093/jxb/ers224 22945939 PMC3467294

[B14] Ludwig-Müller J. (2015). Plants and endophytes: equal partners in secondary metabolite production? Biotechnol. Lett. 37, 1325–1334. 10.1007/s10529-015-1814-4 25792513

[B15] Navet R. Jarmuszkiewicz W. Almeida A. M. Sluse-Goffart C. Sluse F. E. (2003). Energy conservation and dissipation in mitochondria isolated from developing tomato fruit of ethylene-defective mutants failing normal ripening: the effect of ethephon, a chemical precursor of ethylene. J. Bioenerg. Biomembr. 35, 157–168. 10.1023/A:1023750204310 12887014

[B16] Ouyang Z. Zhang L. Zhao M. Wang P. X. Wei Y. Fang J. (2012). Identification and quantification of sesquiterpenes and polyacetylenes in *Atractylodes lancea* from various geographical origins using GC-MS analysis. J. Pharmacogn. 22, 957–963. 10.1590/S0102-695X2012005000051

[B17] Rahimi S. Kim Y. J. Yang D. C. (2015). Production of ginseng saponins: elicitation strategy and signal transductions. Appl. Microbiol. Biotechnol. 99, 6987–6996. 10.1007/s00253-015-6806-8 26194557

[B18] Ren C.-G. Chen Y. Dai C.-C. (2013). Cross-talk between calcium–calmodulin and brassinolide for fungal endophyte-induced volatile oil accumulation of *Atractylodes lancea* plantlets. J. Plant Growth Regul. 33, 285–294. 10.1007/s00344-013-9370-4

[B19] Ren C. G. Dai C. C. (2012). Jasmonic acid is involved in the signaling pathway for fungal endophyte-induced volatile oil accumulation of *Atractylodes lancea* plantlets. BMC Plant Biol. 12:128. 10.1186/1471-2229-12-128 22856333 PMC3681289

[B20] Ren C. G. Dai C. C. (2013). Nitric oxide and brassinosteroids mediated fungal endophyte-induced volatile oil production through protein phosphorylation pathways in *Atractylodes lancea* plantlets. J. Integr. Plant Biol. 55, 1136–1146. 10.1111/jipb.12087 23773784

[B21] Steffens B. (2014). The role of ethylene and ROS in salinity, heavy metal, and flooding responses in rice. Front. Plant Sci. 5:685. 10.3389/fpls.2014.00685 25538719 PMC4255495

[B22] Verhage A. van Wees S. C. Pieterse C. M. (2010). Plant immunity: it's the hormones talking, but what do they say? Plant Physiol. 154, 536–540. 10.1104/pp.110.161570 20921180 PMC2949039

[B23] Wang H. X. Liu C. M. Liu Q. Gao K. (2008). Three types of sesquiterpenes from rhizomes of *Atractylodes lancea*. Phytochemistry 69, 2088–2094. 10.1016/j.phytochem.2008.04.008 18511090

[B24] Wang X. M. Yang B. Ren C. G. Wang H. W. Wang J. Y. Dai C. C. (2015a). Involvement of abscisic acid and salicylic acid in signal cascade regulating bacterial endophyte-induced volatile oil biosynthesis in plantlets of *Atractylodes lancea*. Physiol. Plant. 153, 30–42. 10.1111/ppl.12236 24862990

[B25] Wang X. M. Yang B. Wang H. W. Yang T. Ren C. G. Zheng H. L.. (2015b). Consequences of antagonistic interactions between endophytic fungus and bacterium on plant growth and defense responses in *Atractylodes lancea*. J. Basic Microbiol. 55, 659–670. 10.1002/jobm.201300601 24293321

[B26] Wang Y. Dai C. C. Cao J. L. Xu D. S. (2012). Comparison of the effects of fungal endophyte *Gilmaniella* sp. and its elicitor on Atractylodes lancea plantlets. World J. Microbiol. Biotechnol. 28, 575–584. 10.1007/s11274-011-0850-z 22806853

[B27] Wang Y. Dai C.-C. Zhao Y.-W. Peng Y. (2011). Fungal endophyte-induced volatile oil accumulation in *Atractylodes lancea* plantlets is mediated by nitric oxide, salicylic acid and hydrogen peroxide. Process Biochem. 46, 730–735. 10.1016/j.procbio.2010.11.020

[B28] Wangchuk P. Tobgay T. (2015). Contributions of medicinal plants to the Gross National Happiness and Biodiscovery in Bhutan. J. Ethnobiol. Ethnomed. 11, 48. 10.1186/s13002-015-0035-1 26037080 PMC4469394

[B29] Wei L. J. Deng X. G. Zhu T. Zheng T. Li P. X. Wu J. Q.. (2015). Ethylene is involved in brassinosteroids induced alternative respiratory pathway in cucumber (*Cucumis sativus* L.) seedlings response to abiotic stress. Front. Plant Sci. 6:982. 10.3389/fpls.2015.00982 26617622 PMC4639706

[B30] Yang T. Du W. Zhou J. Wang X.-X. Dai C.-C. (2013). Effects of the symbiosis between fungal endophytes and *Atractylodes lancea* on rhizosphere and phyllosphere microbial communities. Symbiosis 61, 23–36. 10.1007/s13199-013-0254-y

[B31] Yang T. Ma S. Dai C. C. (2014). Drought degree constrains the beneficial effects of a fungal endophyte on *Atractylodes lancea*. J. Appl. Microbiol. 117, 1435–1449. 10.1111/jam.12615 25080260

[B32] Zhao J. Zheng S. H. Fujita K. Sakai K. (2004). Jasmonate and ethylene signalling and their interaction are integral parts of the elicitor signalling pathway leading to beta-thujaplicin biosynthesis in *Cupressus lusitanica* cell cultures. J. Exp. Bot. 55, 1003–1012. 10.1093/jxb/erh127 15047767

[B33] Zhou J. Y. Yuan J. Li X. Ning Y. F. Dai C. C. (2015). Endophytic bacterium-triggered reactive oxygen species directly increase oxygenous sesquiterpenoid content and diversity in *Atractylodes lancea*. Appl. Environ. Microbiol. 82, 1577–1585. 10.1128/AEM.03434-15 26712554 PMC4771314

[B34] Zhou J. Y. Zhao X. Y. Dai C. C. (2014). Antagonistic mechanisms of endophytic *Pseudomonas fluorescens* against *Athelia rolfsii*. J. Appl. Microbiol. 117, 1144–1158. 10.1111/jam.12586 24962812

