# Supplementary figures and images for: The Mechanism of Ethylene Signaling Induced by Endophytic Fungus Gilmaniella sp. AL12 Mediating Sesquiterpenoids Biosynthesis in Atractylodes lancea
Source: Front Plant Sci. 2016 Mar 23;7:361. doi: 10.3389/fpls.2016.00361 (PMC4804159; doi:10.3389/fpls.2016.00361)

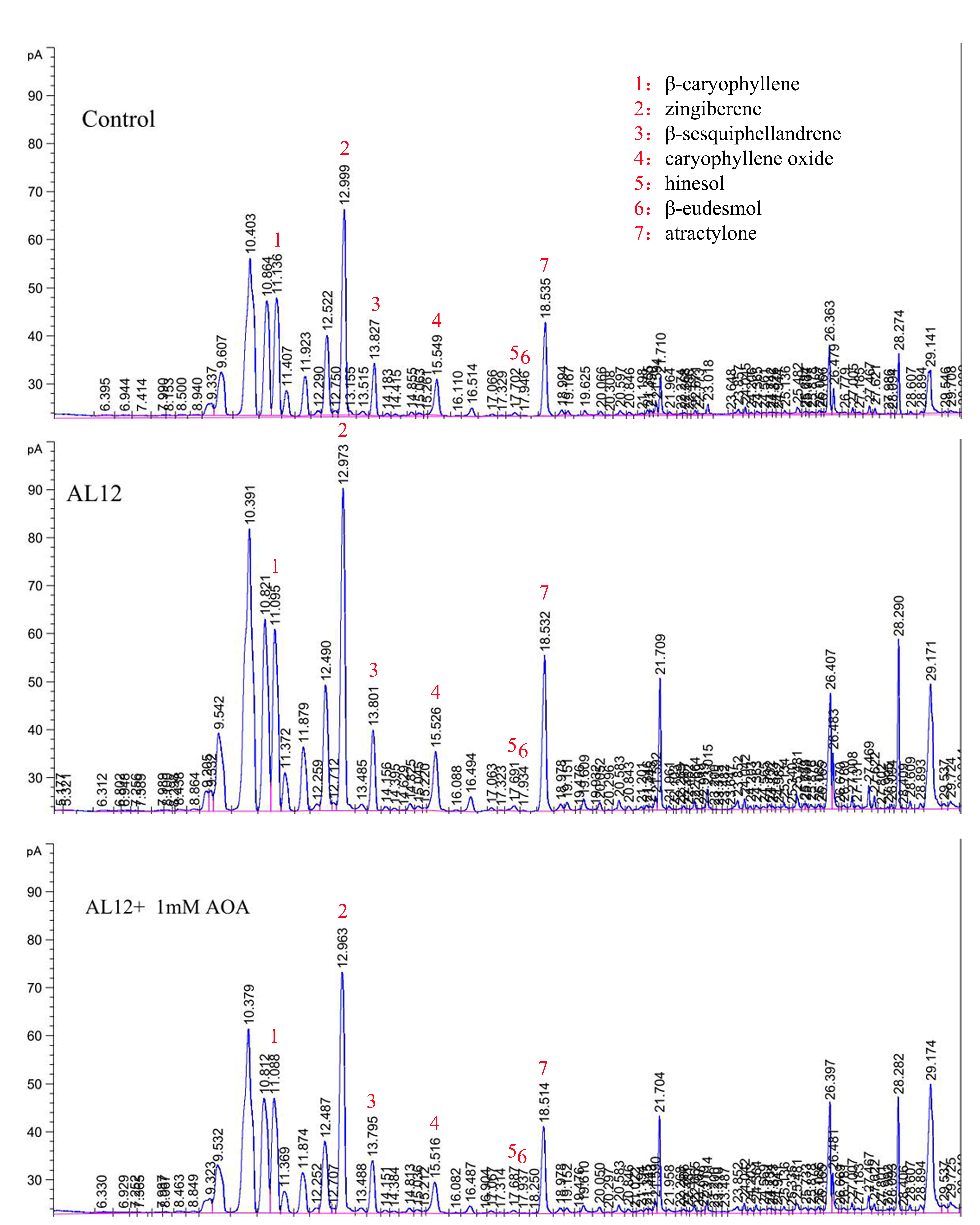

Supplement: Supplementary Figure S1 — Gas chromatograph analysis of sesquiterpenoids in Atractylodes lancea. [file Image1.JPEG]
